# Evaluation of the risk of occupational exposure to antineoplastic drugs in healthcare sector: part II – the application of the FMECA method to compare manual vs automated preparation

**DOI:** 10.2478/aiht-2024-75-3803

**Published:** 2024-03-29

**Authors:** Stefano Dugheri, Giovanni Cappelli, Donato Squillaci, Ilaria Rapi, Niccolò Fanfani, Fabrizio Dori, Michele Cecchi, Viola Sordi, Andrea Ghiori, Nicola Mucci

**Affiliations:** University of Florence, Department of Experimental and Clinical Medicine, Industrial Hygiene and Toxicology Laboratory, Florence, Italy; AOU Meyer, Health and Safety Service, Florence, Italy; Careggi University Hospital, Pharmacy AD Preparation Unit, Florence, Italy

**Keywords:** acceptable risk levels, failure mode effects and criticality analysis, measured risk levels, risk priority number, prihvatljive razine rizika, analiza pogrešaka i kritičnosti posljedica, izmjerene razine rizika, ocjena prioriteta rizika

## Abstract

Healthcare workers handling antineoplastic drugs (ADs) in preparation units run the risk of occupational exposure to contaminated surfaces and associated mutagenic, teratogenic, and oncogenic effects of those drugs. To minimise this risk, automated compounding systems, mainly robots, have been replacing manual preparation of intravenous drugs for the last 20 years now, and their number is on the rise. To evaluate contamination risk and the quality of the working environment for healthcare workers preparing ADs, we applied the Failure Mode Effects and Criticality Analysis (FMECA) method to compare the acceptable risk level (ARL), based on the risk priority number (RPN) calculated from five identified failure modes, with the measured risk level (MRL). The model has shown higher risk of exposure with powdered ADs and containers not protected by external plastic shrink film, but we found no clear difference in contamination risk between manual and automated preparation. This approach could be useful to assess and prevent the risk of occupational exposure for healthcare workers coming from residual cytotoxic contamination both for current handling procedures and the newly designed ones. At the same time, contamination monitoring data can be used to keep track of the quality of working conditions by comparing the observed risk profiles with the proposed ARL. Our study has shown that automated preparation may have an upper hand in terms of safety but still leaves room for improvement, at least in our four hospitals.

## Abstract (hr)

Zdravstveni radnici koji rukuju antineoplastičnim lijekovima u posebnim jedinicama za njihovu pripremu izlažu se zagađenim površinama i riziku od s njima povezanim mutagenim, teratogenim i onkogenim učincima. Da bi se smanjio taj rizik, u posljednjih se 20-ak godina ručna priprema intravenskih lijekova sve više zamjenjuje automatiziranom. Da bismo ocijenili rizik od zagađenja i kakvoću radnog okoliša zdravstvenih radnika koji pripremaju antineoplastične lijekove, oslonili smo se na metodu analize mogućih pogrešaka u postupcima i kritičnosti njihovih posljedica (engl. *failure mode effects and criticality analysis*, krat. FMECA) radi usporedbe prihvatljive razine rizika (engl. *acceptable risk level*, krat. ARL), koja je zbroj ocjena prioriteta rizika (eng. *risk priority number*, krat. RPN) pet utvrđenih načina pogreške s izmjerenom razinom rizika (engl. *measured risk level,* krat. MRL). Taj nam je model pokazao da antineoplastični lijekovi u prahu i spremnicima koji nisu zaštićeni vanjskim plastičnim omotačem donose povećani rizik, ali nismo utvrdili dosljednu razliku u riziku od zagađenja između ručne i automatizirane pripreme. Ovaj pristup može biti koristan u procjeni i kontroli rizika od profesionalne izloženosti u zdravstvenih radnika rezidualnim citotoksičnim spojevima, bilo da se radi o postojećim ili tek planiranim/novoosmišljenim postupcima pripreme lijekova. Podatci dobiveni redovitim nadzorom (monitoringom) istodobno mogu poslužiti za praćenje kakvoće radnih uvjeta tako što će se usporediti s predviđenim profilom rizika koji je utvrđen ARL-om. Iako je naše istraživanje pokazalo da automatizirana priprema ima prednost nad ručnom u smislu sigurnosti, prostora za njezino poboljšanje ima podosta, naročito u našim četirima bolnicama koje su sudjelovale u istraživanju.

Due to their low therapeutic index and iatrogenic risk, anticancer drugs (AD) are considered “high alert drugs” (1, 2), and their preparation for intravenous (IV) application is the most labour-intensive activity. Handling cytotoxic agents, which involves compounding, administration, and waste management, poses a considerable hazard to healthcare professionals, such as nurses, pharmacy technicians, pharmacists, and clinicians (3). A series of multicentre studies on AD contamination run by a research team in British Columbia has identified as many as eleven job categories with the potential for dermal exposure to surfaces contaminated with ADs (4), such as exterior surfaces of vials and handling surfaces, including safety cabinets (both the interior and exterior areas). (5, 6). Nowadays, dermal exposure is the main route of exposure to ADs (7). Recently, Korczowska et al. (8) reported that of 560 wipe tests collected from 28 hospital units in 16 European countries 268 were positive (48 %) for ADs, whereas 21 of the 28 (75 %) hospitals had over 30 % of positive samples.

The first report of occupational exposure to cytostatic drugs and the associated health risks appeared in the 1979 study by Falck et al. (9), who analysed their mutagenic activity with the Ames assay in the urine of nurses preparing and administering them without protective measures. In 2000, the Italian Istituto Superiore di Sanità (ISS) found that 19.6 % of nurses had been accidentally contaminated with ADs in preparation and/or administration units (10). Currently, of the 331 oncology units in Italy surveyed by the Italian Society of Hospital Pharmacies (Società Italiana di Farmacia Ospedaliera e dei Servizi Farmaceutici delle Aziende Sanitarie), about 80 % prepare an average of 20,000 AD doses a year (11), which calls for greater centralisation of AD preparation by accredited units, as it has been shown to improve safety, waste reduction, and economic savings (12). For now, accredited centralised units prepare a minimum of 100 drug doses a day in two 12-hour shifts (13,14,15).

As the increasing demand for ADs puts a stress on the system, automation has offered a safer and less error-prone alternative to manual preparation. The use of robots in chemotherapy, in fact, is not recent and dates back to the 1989 (16). Surprisingly, though, automation has not yet become common. The first modern drug-compounding robot (IntelliFill IV) was introduced by ForHealth Technologies in 2002 and has prepared over 24 million doses to date. Soon it was followed by the RIVA robot (Intelligent Hospital Systems) and the CytoCare robot (Health Robotics) (17), yet there are still no specific guidelines for automated compounders, and literature on qualification and validation methodologies is scarce (3, 18, 19, 20). Recently, the United States Pharmacopeia (USP) regulations have given serious consideration to evaluating this technology. The worldwide adoption of robotic compounding in oncology centres and hospitals has led to significant improvements in the use and functionality of these devices in the past decade (3, 21, 22, 23). Automated systems have a maximum annual capacity of about 50,000 preparations with a preparation time of up to 10 min and cost of 19.18 euros, whereas manual preparation costs about 25.70 euros (24).

In any case, both manual and automated preparation of ADs are subject to errors and failures that can result in contamination and put workers’ health at risk. These concerns have prompted various countries and organisations to develop guidelines for correct AD handling (25,26,27). To identify hazards and assess risks, recent ones promote utilising methods such as the Failure Mode, Effects and Criticality Analysis (FMECA). FMECA was created by the Grumman Aircraft Corporation for the NASA’s Apollo programme and is extensively used in industries such as defence, shipbuilding, medical, and insurance to gauge the safety of systems and the reliability of processes and designs (28). This kind of risk mapping is based on the so-called risk priority number (RPN), an index rating the importance of each critical step of the processes to prioritise corrective action and achieve greater efficiency over time.

In recent years, FMECA has gained momentum in the pharmaceutical and healthcare sectors for the purposes of healthcare management. It is presently endorsed by the US Food and Drug Administration (FDA) to reduce medication errors and is one of the foremost systematic methodologies for failure analysis (29, 30). In the field of industrial hygiene, it is a reference method for risk assessment (31,32,33) and provides a solid basis to improve occupational safety and contamination control.

The aim of our study was to apply FMECA to evaluate surface contamination with ADs and the quality of working environment and to assess the risk of occupational exposure for healthcare workers during either manual or automated (robotic) preparation of ADs. To do that we adopted the RPN rating to establish an acceptable risk level (ARL) and compare it with the results obtained from environmental monitoring, expressed as measured risk level (MRL).

## MATERIALS AND METHODS

### FMECA method

**Figure 1:**
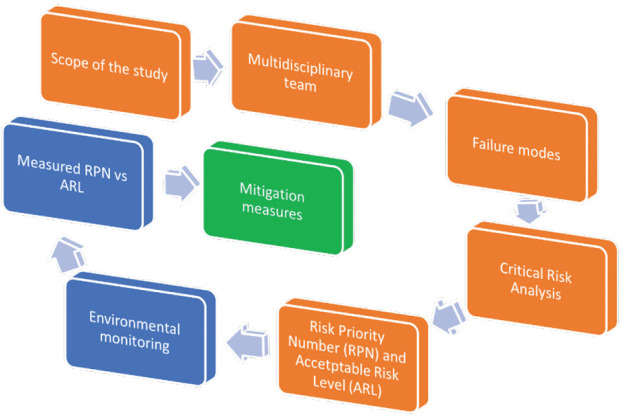
FMECA analysis and the Acceptable Risk Level estimation process

The FMECA method consists of five steps: i) specifying the study’s extent, ii) constituting a multidisciplinary team, iii) detecting failure modes that could affect the procedure, gathering information about the procedure, iv) running criticality analysis to prioritise potential failure modes, and v) proposing corrective action and outcome measures to mitigate risks (Figure 1).

Our team consisted of three pharmacists, a risk manager, and three laboratory technicians with expertise in monitoring drug contamination. Based on multidisciplinary experience and drug information (Table 1), we identified eight main AD groups: i) available only in powdered form, ii) available only in liquid form, iii) corrosive, iv) available in one concentration only, v) unstable, vi) without plastic shrink or break-proof container (no holder casing), vii) requiring time for reconstitution or with tendency to crystallise (poorly soluble), and viii) those with higher average therapeutic concentrations.

**Table 1:** Main characteristics of antineoplastic drugs evaluated in the FMECA study (data obtained from the Agenzia Italiana del Farmaco database)

**ID substance**	**Trade packaging**	**Volume packaging–liquid (mg/mL)**	**Reconstituted concentration–powder mg/mL)**	**Recommended dosage**	**Corrosive**	**Stability**	**Poorly soluble**	**Packaging**
MITC	10 mg to 40 mg		0.5 or 1	10–20 mg/m^2^		Immediate use		Glass
DC	100 mg to 1 g		1.4–2.0 or 2.8–4.0	200–250 mg/m^2^		No information		Glass
RTX	2 mg		0.04–0.008	3 mg/m^2^		Up to 12 h		Glass
FTM	208 mg		52	100 mg/m^2^		Immediate use		Glass
DNR	20 mg		2	0.5–3 mg/kg.		Up to 24 h at 20–25 °C or 48 h at 2–8 °C		Glass
VNB	10 mg		1	3.7 mg/m^2^		Up to 28 days at 2–8 °C		Glass
MP	2 mg to 200 mg		5	8–200 mg/m^2^		Immediate use or up to 1.5 h at 20–25 °C	x	Glass
VNC	1 mg to 5 mg		1	0.4–1.4 mg/m^2^		No information		Glass, PP+plastic
CP	200 mg to 1 g		20	12–240 mg/m^2^			x	Type III glass
PMX	100 mg to 1 g		25	500 mg/m^2^		Up to 24 h at 2–8 °C		Glass
TPT	1 mg to 4 mg		1	1.5 mg/m^2^		Up to 24 h at 2–8 °C to 30 days at 25 °C		Glass
EPI	5 mg to 200 mg		2	60–135 mg/m^2^		24 h at 20–25 °C to 7–28 days		Amber glass
DXR	10 mg to 200 mg	2	1–2	50–75 mg/m^2^		24 h at 20–25 °C to 7 days at 25 °C		Glass
IRT	20 mg to 1 g	1.5–20		180 mg/m^2^–350 mg/m^2^		6 h at 20–25 °C to 24 h at 2–8 °C		PP, glass
PTX	30 mg to 600 mg	6	1–5	100 mg/m^2^–260 mg/m^2^	x	4 h at 25 °C to 7 days at 5 °C and 25 °C		Glass or glass+PP
BSF	60 mg	6		0.8–3.2 mg/kg	x	4 h at 20–25 °C to 12 h at 2–8 °C		Glass
Carbo Pt	50 mg to 600 mg	10		400 mg/m^2^		3 h at 15–35 °C to 24 h at 2–8 °C		Amber glass or PP
CisPt	10 mg to 100 mg	0.5–1		50–120 mg/m^2^		6 to 24 h at 20–25 °C		Amber glass or PP
DTX	20 mg to 160 mg	10–20		75 mg/m^2^		6 h under 25 °C to 3 days at 2–8 °C	x	Amber glass or glass
IDC	5 mg and 10 mg		1	12 mg/m^2^		Up to 48 h at 2–8 °C or 24 h at 20–25 °C		Glass
MT	2.5 mg to 0.5 g	7.5–100		10–25 mg/m^2^ or 7.5–25 mg/week		Immediate use or temperature< 25 °C		Glass
5-FU	50 mg to 5 g	40–50		200–600 mg/m^2^ or 12 mg/kg	x	24 h at 25 °C to 48 h		Glass or aluminium with epoxy phenolic lacquer
CTB	100 mg to 5 g	20–100		100–200 mg/m^2^		24 h at <30 °C to 72 h at 2–8 °C	x	Glass
ETP	50 mg to 1 g	20–100		60–200 mg/m^2^		24 h at 20–25 °C to 96 h		Amber glass
OxaliPt	50 mg to 250 mg	5		85 mg/m^2^		up to 48 h at 2–8 °C or 6–24 h at 25 °C		Glass
VNR	10 mg to 80 mg	10 or 20–80		25–80 mg/m^2^		up to 24 h at 2–8 °C or 25 °C		Glass or PVC/PVDC/aluminium

5-FU – 5-fluorouracil; BSF – busulfan; CarboPt – carboplatin; CisPt – cisplatin; CP – cyclophosphamide; CTB – cytarabine; DC – dacarbazine; DNR – daunorubicin; DTX – docetaxel; DXR – doxorubicin; EPI – epirubicin; ETP – etoposide; FTM – fotemustine; GEM – gemcitabine; IDC – idarubicine; IP – iphosfamide; IRT – irinotecan; MITC – mitomycin C; MP – melphalan; MT – methotrexate; OxaliPt – oxaliplatin; PMX – pemetrexed; PP – polypropylene; PTX – paclitaxel; PVC – polyvinyl chloride; PVDC – polyvinylidene chloride; RTX – raltitrexed; TMX – tamoxifen; TPT – topotecan; VNB – vinblastine; VNC – vincristine; VND – vindesine; VNR – vinorelbine

**Figure 2:**
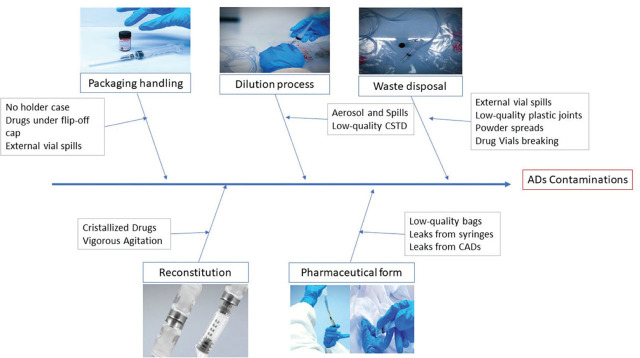
Ishikawa diagram of failure modes

For each group, five potential failure modes were evaluated following the Ishikawa diagram, i.e. five steps of AD preparation that could lead to operator exposure: packaging/handling, reconstitution, dilution, pharmaceutical form [bags, syringes, elastomeric pumps, and central access device (CADs)] and waste disposal (Figure 2).

To quantify the RPNs for the eight AD groups, we defined a five-point scale to score the five failure modes taking into account the severity, potential frequency of occurrence, and the possibility of avoidance. Failure mode scores range from 1 to 5, where 1 corresponds to a very low risk (low severity, low frequency, and completely to avoidable contamination) and 5 to a very high risk (high severity, very high frequency, and unavoidable contamination). Then from the sum of RPNs we defined the ARL, a value that represents acceptable risk in operating conditions, and which ranges from 5 (1+1+1+1+1) to 25 (5+5+5+5+5).

Then, we used the same five-point RPN scale to rate monitored quality of the working environment and the spread of AD contamination in five parameters: the number of ADs found by monitoring, their spread on contaminated surfaces, the percentage of positive wipe samples, their spread on protective gloves (indicating dermal exposure), and the percentage of positive gloves (Table 2).

**Table 2:** Risk priority rating of environmental monitoring findings assessing antineoplastic drug contamination [based on alert glove values from Dugheri et al. (34)]

**RPN**	**Number of detected ADs**	**Surface contamination spread (pg/cm^2^)**	**Percentage of contaminated surfaces (%)**	**Glove contamination spread (pg/cm^2^)**	**Percentage of contaminated gloves (%)**
1 – very low	1	LOQ–10	<3	<LOQ	<2
2 – low	2	11–30	4–6	1/10 of AGV 90^th^ percentile	3–5
3 – moderate	3	31–50	7–9	AGV 90^th^ percentile	6–8
4 – high	4–5	50–99	10–12	1/10 of AGV 95^th^ percentile	8–10
5 – very high	>6	>100	>12	AGV 95^th^ percentile	>10

ADs – antineoplastic drugs; AGV – alert glove value; RPN – risk priority number

**Figure 3:**
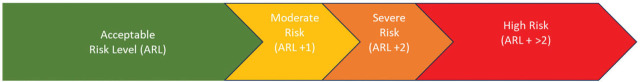
Risk levels strip for MRL comparison with ARL

To profile the risk for the eight AD groups based on monitoring findings we compared the sum of RPNs expressed as MRL with the corresponding ARLs, as follows (Figure 3): MRL=ARL − risk under control; MRL=ARL+1 − moderate risk that should be mitigated; MRL=ARL+2 − severe uncontrolled risk that should be mitigated; and MRL=ARL+>2 − high uncontrolled risk, mitigation mandatory.

This assessment was carried out for both automated and manual processes.

### Environmental monitoring: sample collection and preparation

Surface contamination with ADs was assessed in four Italian hospitals by wipe and glove sampling at the beginning and end of the work shift as described in detail elsewhere (34). Two hospitals had manual and two robotic systems, namely the APOTECAchemo (Loccioni Humancare, Ancona, Italy) and IV Station^®^ (Omnicell, Fort Worth, TX, USA) for automated mixing of dangerous drugs for sterile injection. These robots were installed in a separate room with a negative pressure gradient, laminar airflow, and class A air quality (35), detached from the biological safety cabinet. The components for preparation are manually loaded and then transferred by a robot arm. Gravimetric control guarantees precise drug dosing, and all personnel actions, time stamps, drug labels, input and output materials are recorded to ensure traceability.

Manual preparation, in turn, is carried out by two technicians in a separate clean room with a class II type B3 biological safety cabinet with laminar airflow (36). One technician handles the preparation and the other coordinates it from outside the cabinet and inserts vials. After each operation, a pharmacist checks the final preparations for quality and quantity.

## RESULTS AND DISCUSSION

Table 3 shows the assessed ARLs for each AD group. Packaging, handling, and waste disposal are the failure modes that received the highest RPN for all AD groups, and dilution the lowest. Powdered ADs, ADs without holder casing, and ADs with high average therapeutic concentration were estimated to carry the highest risk. Automatic preparation was estimated to carry lower risk than the manual one (lower ARL) for all the eight AD groups.

**Table 3:** Evaluation of five failure modes with risk priority numbers (RPNs) for each antineoplastic drug group in FMECA analysis

**Failure mode**	**Powder ADs**		**Liquid ADs**		**Corrosive ADs**		**One-concentration ADs**		**Unstable ADs**		**No holder casing**		**High average therapeutic concentration**		**Poorly soluble**	
	**MITC, DC, DNR, IP, FTM, VNB, GEM, CP, MP, VND, PMX, TPT, RTX**		**VNC, EPI, DXR, IRT, 5-FU, PTX, BSF, CarboPt, CisPt, CTB, DTX, ETP, GEM, IDC, MT, OxaliPt, PMX, VNR**		**BSF, TX, 5-FU**		**RTX, FTM, DNR, VNB, BSF**		**DXR, FTM, MP, MITC, VNC, MT**		**VND, VNC, VNR, TPT, RTX, MITC, MP, IP, IDC, DXR, EPI, CP, CarboPt**		**CP, IP, DC, GEM, MP, PMX, IRT, PTX, 5-FU**		**5-FU, CP**	
	**RPN**		**RPN**		**RPN**		**RPN**		**RPN**		**RPN**		**RPN**		**RPN**	
	**M**	**A**	**M**	**A**	**M**	**A**	**M**	**A**	**M**	**A**	**M**	**A**	**M**	**A**	**M**	**A**
Packaging handling	4	4	2	2	4	4	3	3	3	3	5	5	3	3	3	3
Reconstitution	5	4	1	1	2	1	4	4	3	2	3	2	4	3	4	3
Dilution process	3	2	4	4	2	1	4	4	3	2	3	2	4	2	4	3
Pharmaceutical form	2	2	3	2	3	3	3	2	3	3	2	2	4	4	3	3
Waste disposal	3	3	3	3	5	3	3	2	4	4	5	5	2	2	2	2
**Acceptable risk level (ARL)**	**17**	**15**	**13**	**12**	**16**	**12**	**17**	**15**	**16**	**14**	**18**	**16**	**17**	**14**	**16**	**14**

M=manual preparation; A= automated preparation. The Acceptable Risk Level (ARL) is the sum of RPNs. 5-FU – 5-fluorouracil; BSF – busulfan; CarboPt – carboplatin; CisPt – cisplatin; CP – cyclophosphamide; CTB – cytarabine; DC – dacarbazine; DNR – daunorubicin; DTX – docetaxel; DXR – doxorubicin; EPI – epirubicin; ETP – etoposide; FTM – fotemustine; GEM – gemcitabine; IDC – idarubicine; IP – iphosfamide; IRT – irinotecan; MITC – mitomycin C; MP – melphalan; MT – methotrexate; OxaliPt – oxaliplatin; PMX – pemetrexed; PTX – paclitaxel; RTX – raltitrexed; TMX – tamoxifen; TPT – topotecan; VNB – vinblastine; VNC – vincristine; VND – vindesine; VNR – vinorelbine

Table 4 shows the obtained MRLs from monitoring and the assessed risk with respect to ARLs. Most AD groups had higher MRLs than their respective ARLs, save for liquid, unstable ADs, and one-concentration ADs in automated preparation, and for corrosive and unstable ADs in manual preparation. The highest risk was determined for powdered ADs in both manual and automated preparation and for ADs without a holder casing in manual preparation. Contrary to the FMECA ARL estimates, automated preparation did not always have lower MRLs than the manual one.

**Table 4:** Evaluation of five failure modes with risk priority numbers (RPNs) related to environmental monitoring data

**Failure**	**Powder ADs**		**Liquid ADs**		**Corrosive ADs**		**One-concentration ADs**		**Unstable ADs**		**No holder casing**		**High average therapeutic concentration**		**Poorly soluble**	
	**MITC, DC, DNR, IP, FTM, VNB, GEM, CP, MP, VND, PMX, TPT, RTX**		**VNC, EPI, DXR, IRT, 5-FU, PTX, BSF, CarbPt, CisPt, CTB, DTX, ETP, GEM, IDC, MT, OxaliPt, PMX, VNR**		**BSF, PTX, 5-FU**		**RTX, FTM, DNR, VNB, BSF**		**DXR (Miocet), FTM, MP, MITC,**		**VND, VNC, VNR, TPT, RTX, MITC, MP, IP, IDC, DXR, EPI, CP, CarboPt**		**CP, IP, DC, GEM, MP, PMX, IRT, PTX, 5-FU**		**5-FU, CP**	
	**RPN**		**RPN**		**RPN**		**RPN**		**RPN**		**RPN**		**RPN**		**RPN**	
	**M**	**A**	**M**	**A**	**M**	**A**	**M**	**A**	**M**	**A**	**M**	**A**	**M**	**A**	**M**	**A**
Number of detected ADs	3	3	3	3	1	1	2	2	2	2	3	2	3	3	2	2
Surface contamination spread	5	5	3	3	5	5	4	3	4	3	5	4	5	5	5	5
Percentage of contaminated surfaces	3	2	3	3	1	2	4	4	3	3	4	4	4	3	3	3
Glove contamination spread	4	4	3	1	4	4	4	3	3	4	4	3	3	3	3	2
Percentage of contaminated gloves	5	5	2	1	2	1	2	2	2	2	5	5	3	2	5	4
Measured risk level (MRL)	20	19	14	11	15	13	18	14	14	14	21	18	18	16	18	16
Acceptable risk level (ARL, Table 3)	17	15	13	12	16	12	17	15	16	14	18	16	17	14	16	14
Assessed risk	High	High	Moderate	Moderate	Moderate	Moderate	Moderate	Moderate	Moderate	Moderate	High	Severe	Moderate	Severe	Severe	Severe

The measured risk level (MRL) is the sum of RPNs. Acceptable risk level is taken from Table 3. M=manual preparation; A= automated preparation. 5-FU – 5-fluorouracil; BSF – busulfan; CarboPt – carboplatin; CisPt – cisplatin; CP – cyclophosphamide; CTB – cytarabine; DC – dacarbazine; DNR – daunorubicin; DTX – docetaxel; DXR – doxorubicin; EPI – epirubicin; ETP – etoposide; FTM – fotemustine; GEM – gemcitabine; IDC – idarubicine; IP – iphosfamide; IRT – irinotecan; MITC – mitomycin C; MP – melphalan; MT – methotrexate; OxaliPt – oxaliplatin; PMX – pemetrexed; PTX – paclitaxel; RTX – raltitrexed; TMX – tamoxifen; TPT – topotecan; VNB – vinblastine; VNC – vincristine; VND – vindesine; VNR – vinorelbine

A proactive method such as FMECA has already been used to assess the risks involved in drug preparation (37, 38), and we have found it quite useful for our study. We expanded the potential failure modes (or steps) described by Spivey and Connor (38) with waste disposal due to the well-known contamination of AD vials on the outside (39,40,41). Our MRLs confirm this risk with higher RPNs for ADs without holder casing, exceeding their respective ARLs by 3 for manual (high risk) and 2 for automated preparation (severe risk). Handling glass vials without a plastic shrink requires frequent changing of gloves and decontamination of the external vial surface every time a new package is opened. In addition, to minimise exposure risk during waste disposal, plastic bags (minimum 2 mm thick if polypropylene or 4 mm if polyethylene) should be used to gather potentially contaminated materials. All workplaces should have a policy for the isolation of waste materials resulting from cytotoxic drug preparation. In case of accidental spills due to vial breaks, the area should be marked with a caution sign and cleaned by trained staff. Lastly, the preparation should be done by as few people as possible, but two is the minimum.

Concerning reconstitution, both ADs in powdered form and those available in one concentration alone had a high ARL (17 for manual and 15 for automated), reflecting higher exposure risk while accessing the vial with a syringe or using closed system drug transfer devices (CSTDs) to add a solubilising liquid vehicle, as the procedure may stir small quantities of ADs to become airborne and spread in the environment. Spread and inhalation is a concern even with good practices and personal protection equipment in place (42). Our monitoring findings confirm these concerns, as powdered ADs, which always require reconstitution, had MRLs higher than respective ARLs by 3 and 4, respectively (high risk) for both manual and automated processes. Cotteret et al. (43) point to a significant contamination with drugs adhering to the flip-off caps. In our previous study (44), we too have identified powdered ADs as a major source of contamination: the three most frequently detected substances on surfaces and gloves were cyclophosphamide (13.5 %), gemcitebine (9.4 %), and iphosfamide (6.5 %). Moreover, powdered cyclophosphamide is reconstituted with vigorous shaking, and it takes up to 30 min for it to completely dissolve (45). It is perhaps for this reason that CP-containing AD groups (powdered ADs, no holder casing, poorly soluble) have higher RPN for the percentage of contaminated gloves (RPN 5 corresponds to >10 % glove contamination). To reduce the risk linked to reconstitution and dissolution, the US FDA has therefore approved a new ready-to-dilute, 200 mg/mL vial for cyclophosphamide injection in 2023 (46).

Our FMECA analysis has also identified dilution and pharmaceutical forms, such as bags, syringes, elastomeric pumps, and central access devices as entailing increased risk of contamination. Liquid ADs, ADs available in one concentration, and those used in high concentrations had the highest RPN. Liquid ADs, most notably those available in one concentration only, often require several dilution steps to meet the required dose for a specific patient (47), which increases the risk of aerosol generation and spills. Furthermore, ADs in high average therapeutic concentrations present an even greater risk as micro-spills are likely to be highly concentrated.

Surface contamination spread and percentages of contaminated surfaces varied in RPN across AD groups, but surface contamination spread was rated high for powdered ADs, corrosive ADs, those without holder casing in manual prep, highly concentrated ADs, and the poorly soluble ones (RPN 5, which corresponds to >100 pg/cm^2^) (Table 4). There are many kinds of CSTDs made of different plastic materials. Our monitoring experts reported that some CSTD junctions presented micro-spills during the use of corrosive ADs such as busulfan or paclitaxel. This is the reason why the corrosive ADs group received high RPN in terms of surface contamination.

Concerning the comparison between manual and automated preparation, our FMECA analysis finds the latter less risky due to reduced operator contact with drugs. This is why modern robots account for 65 % of the total AD preparation today (24). However, our monitoring RPNs show no consistent risk reduction by automated vs manual preparation, especially in summary MRLs, probably due to the high level of training and use of suitable protective equipment and closed systems in participating hospitals. In addition, automated systems have been introduced only recently in these hospitals, and it may take more time to fully benefit from them. Additional research is therefore needed to evaluate the benefits of robotic AD compounders for worker safety.

## CONCLUSION

We have found FMECA a valuable method to identify and rank potential weaknesses in cytotoxic drug preparation, whether existing or being developed for future use. Combined with regular contamination monitoring to compare the current risk with the proposed acceptable one, it can single out corrective actions to improve safety culture and increase staff vigilance.

Our study has also shown that automated preparation may have an upper hand in terms of safety but still leaves room for advancement, at least in our four hospitals. With budget constraints imposed on hiring staff and the need for fully tracked AD preparation and administration, workflows need constant review and innovative streamlining solutions.
